# *Corynebacterium silvaticum* in a game-handling butcher: A new zoonotic pathogen of human infections

**DOI:** 10.1016/j.idcr.2026.e02610

**Published:** 2026-05-14

**Authors:** Andreas Sing, Alexandra Dangel, Vyacheslav G. Melnikov, Hildegard Angermeier, Katja Bengs, Robert B. Brauer, Anja Berger

**Affiliations:** a German National Consiliary Laboratory for Diphtheria, WHO Collaborating for Diphtheria, Bavarian Health and Food Safety Authority, EU Reference Laboratory for Public Health on Diphtheria and Pertussis (EURL-PH-DIPE), Veterinärstraße 2, Oberschleißheim 85764, Germany; b NGS Core Unit, Dept. of Public Health Microbiology, Bavarian Health and Food Safety Authority, Veterinärstraße 2, Oberschleißheim 85764, Germany; c German National Consiliary Laboratory for Diphtheria, Veterinärstraße 2, Oberschleißheim 85764, Germany; d Dept. of Bacteriology, Bavarian Health and Food Safety Authority, Veterinärstraße 2, Oberschleißheim 85764, Germany; e Marienkrankenhaus Cochem Chirurgie I, Allgemein, und Viszeralchirurgie, Spezielle Viszeralchirurgie, Proktologie und Unfallchirurgie/Orthopädie, Avallonstraße 32, Cochem 56812, Germany

**Keywords:** *Corynebacterium silvaticum*, Diphtheria, *Corynebacterium diphtheriae* species complex, Zoonosis

## Abstract

The recently described *Corynebacterium (C.) silvaticum* is a diphtheria toxin (*tox*-) gene bearing species so far mainly found in wild boar and deers. Due to its close relationship to *C. ulcerans* a zoonotic potential has been proposed. To date, only two cases of human *C. silvaticum* infections have been reported in the literature. Here we present the case of a 63-year-old male game-hunting butcher with recurrent and difficult-to-treat axillary lymphadenitis and abscess formation. This case underlines the zoonotic potential of this novel member of the *C. diphtheriae* species complex and suggests zoonotic transmission from wild boars via direct contact to infectious tissue and possible microtrauma lesions during unprotected animal processing.

## Highlights

• The third case of human *Corynebacterium silvaticum* underlines its zoonotic potential.
• Game contact probably main risk factor for human *C. silvaticum* infection.
• Molecular phylogeny of all known *C. silvaticum* isolates (animal & human).
• Diagnostic hints for *C. silvaticum* identification (esp. vs. *C. ulcerans*).

## Introduction

Diphtheria is caused by toxigenic strains of *Corynebacterium* spp. that produce the major pathogenicity factor diphtheria toxin (DT). DT is encoded on the *tox* gene, located either on a bacteriophage or within a pathogenicity island [1], [2]. DT is responsible for causing the local respiratory or skin symptoms of diphtheria, i.e. classical respiratory diphtheria with a clinical spectrum from tonsillitis to severe suffocating pharyngitis or cutaneous diphtheria mostly presenting as an acute or chronic skin ulceration. DT is also responsible for the systemic neurological and cardiac symptoms associated with diphtheria [3], [4]. The three species capable of producing DT include the primarily human pathogen *C. diphtheriae* and the zoonotic pathogens *C. ulcerans* and *C. pseudotuberculosis*. In recent years, the *C. diphtheriae* species complex (CdSC) has been expanded mainly based on genomic data, sometimes supported by biochemical properties [5]. Two closely related species were separated from *C. ulcerans*: *C. silvaticum* and *C. ramonii* (formerly known as lineage 2 of *C. ulcerans*).

In 2020, the previously called “*Corynebacterium ulcerans* wildlife cluster” was re-classified as a novel species named *C. silvaticum* causing caseous lymphadenitis in 33 wild boars and a roe deer in Germany [6], [7], [8]. Subsequently, *C. silvaticum* was also detected in an additional wild boar from Germany [9] and a livestock pig from Portugal [10]. So far, all animal isolates from Germany were non-toxigenic, *tox*-bearing (NTTB), while isolates from Portugal were found to be *tox*-positive and isolates from Austria were either NTTB or *tox*-positive [11]. This has led to the proposal of two distinct clades based on molecular characteristics of the *tox* gene: the described isolates from Austria (n = 1) and Portugal (n = 8) belong to Clade 1 and have a complete *tox*-gene, while all known isolates from Germany (n = 39) so far, as well as one from Switzerland and one from Austria, belong to Clade 2 with a GG-insertion after position 46 in the *tox*-gene thus leading to a frameshift mutation resulting in an NTTB status [11].

Since *C. ulcerans*, from which *C. silvaticum* was separated, has previously been described as zoonotic pathogen transmitted from pigs to humans [12], [13] and *C. silvaticum* so far was mainly isolated from diseased or dead wild boars and a domestic pig, it was speculated that *C. silvaticum* might also be a zoonotic pathogen infecting humans. Very recently, two human *C. silvaticum* infections causing axillary lymphadenitis in two males from Northern Bavaria and Lower Saxony in Germany in 2018 and 2021, respectively, were reported [14], one of them being a butcher. Here we describe a third human *C. silvaticum* infection leading to recurrent and difficult-to-treat axillary lymphadenitis and abscess formation in a game-handling butcher.

## Case report

**Fig. 1:**
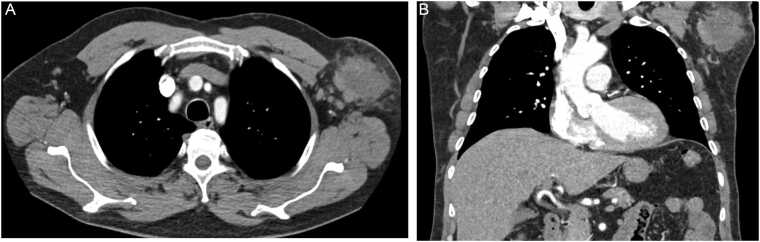
a,b: Computed tomography (CT) scans of the axilla suggesting a 6–7 cm lymphoma in the left axilla.

**Fig. 2:**
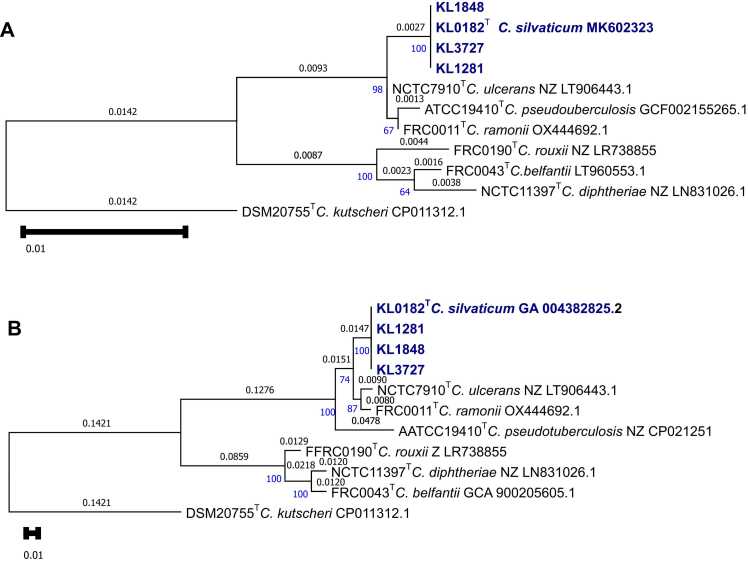
Maximum likelihood gene phylogenies of *16S* (A) and *rpoB* (B) of KL3727 and the previousely described human *C. silvaticum* cases (KL1281 and KL1848) in comparison to *16S* or *rpoB* sequences from publicly available type strain sequences of *CdSC* and a close relative outgroup *(C. kutscheri).* Substitutions per site are indicated in black above branches and local supporting values in blue at intersections of branches. Scale bars indicate number of substitutions per site.

In 2025, a 63-year-old butcher from Rhineland-Palatinate reports frequent finger injuries, occurring particulary during butchering wild boar, when he injures himself on the boar´s tusks. He did not wear protective gloves. A few weeks ago, he noticed increasing swelling in his left axilla. His family doctor ordered a computed tomography (CT) scan (Figs. 1a and 1b) of the axilla. Based on the imaging findings, the radiologist strongly suspected a 6–7 cm lymphoma in the left axilla. Based on the differential blood count (leukocytes 10640/µl, neutrophils 68,1%, eosinophils 1%, monocytes 11,5%, lymphocytes 18,1%), the oncologist was able to rule out a lymphoma and made a presumptive diagnosis of an axillary abscess. Four days later the patient presented at the surgical clinic with a painful 5–7 cm swelling in the left axilla with normal leukocyte count (8700/µl) and slightly elevated CRP levels (3,8 mg/dl). On the same day, the first operation was performed with incision and drainage of the abscess and removal of app. 50 ml of purulent discharge. A microbiological swab and pathological routine examination were ordered for further evaluation. Empirical oral antibiotic therapy with cefuroxime (3x500mg p.o.) was given for 14d along with open wound management according to the hospital´s routine recommendations for antibiotic treatment of suspected mixed soft tissue infections. Despite open wound management, the abscess did not heal. The patient returned five months later with normal leukocytes counts (7600/ µl) to the surgery department. After another CT-scan, that showed a residual abscess with multiple lymph nodes, the lymph node conglomerate (7.5 cm×4 cm x 4 cm) with the residual abscess and superficial skin ulcerations was completely exstirpated. The subsequent course was uneventful, and the axillary wound healed during the 8-month follow up period. Tissue material grew small white relatively dry colonies on blood agar after 48 h at 37°C in pure culture, matrix-assisted laser desorption/ionization time-of-flight mass spectrometry (MALDT-TOF MS) at a primary laboratory identified the strain as *C. ulcerans*. It was sent to the German National Consiliary Laboratory for Diphtheria (GNCLD) for further identification and toxigenicity testing, since toxigenic *Corynebacterium* spp. are legally notifiable in Germany according to the Infection Protection Act. The strain, now named KL3727, was found to ferment glucose, ribose and maltose (like *C. ulcerans*), but not D-xylose, mannitol, lactose, sucrose or glycogen (like *C. pseudotuberculosis*). Interestingly, the strain did initially not grow on a modified Clauberg agar (Xebios Diagnostics, Germany) for 48 h. Sparse growth was observed after prolonged incubation for 72 h, as it had been also seen in the strains KL1281, KL1848 and the type strain *C. silvaticum* sp. nov. DSM 109166T (LMG 31313T, CIP 111672T). MALDI-TOF MS (Bruker, https:www.bruker.com) using the current commercial MBT_K_ database would misidentify *C. silvaticum* as *C. ulcerans*. Using the GNCLD´s expanded database with according reference spectra available on the MALDI-UP user platform (http://maldi-up.ua-bw.de/ (9; 14) the strain was identified as *C. silvaticum* (score value 2.51 and 2.27, respectively, followed by 2.13 and 1.94 for *C. ulcerans*). A new publication by Rau et al. shows the potential to identify members of the CdSC including *C. silvaticum* using the current MALDI-UP version [17]. Next generation sequencing (NGS) analyses, including average nucleotide identity (ANI), *16S* and *rpoB* gene phylogenies, Multi locus Sequence Typing (MLST), core genome (cg)MLST and *tox* gene alignment were performed as described in [14]. The thereby included ANI analysis using MUMmer algorithm showed highest identities for *C. silvaticum* KL0182^T^, well above the species boundary of 96%, whereas identity values with all other species type strains were below 91% (Table 1).In *16S* and *rpoB* gene phylogenies KL3727 shares the branch with the *C.silvaticum* type strain and the two previously published human isolates, with clear separation from the other branches of the other species` type strains (Fig. 2). MLST revealed sequence type (ST) 578, so far exclusively found in *C. silvaticum*. Hence the NGS analyses confirmed the identification as a *C. silvaticum* strain.

**Table 1:** Average nucleotide identity (ANI) values of KL3727 in comparison to the type strain genomes of the CdSC species.

**Type strain**	**species**	**Identity values KL3727**
NCTC11397^T^	*C. diphtheriae*	85.256%
FRC0043^T^	*C. belfantii*	86.173%
FRC0190^T^	*C. rouxii*	86.071%
NCTC7910^T^	*C. ulcerans*	90.849%
FRC0011^T^	*C. ramonii*	90.990%
KL0182^T^	*C. silvaticum*	99.995%
ATCC19410^T^	*C. pseudotuberculosis*	86.059%

Using *tox*-PCR, an optimized immunoprecipitation Elek test, a lateral flow immunoassay (LFIA) and NGS analysis as reported previously [14], the strain was identified as NTTB (i.e. *tox*-PCR positive, Elek/LFIA -test negative). As *C. silvaticum* belongs to the *C. diphtheriae* species Complex (CdSC) antimicrobial susceptibility testing was performed via agar diffusion test and determination of disc diffusion diameters (DDD) according to the EUCAST guidelines for *C. diphtheriae* and *C. ulcerans* (v. 14.0, valid from 2024 to 01–01; https://www.eucast.org/clinical_breakpoints). The DDDs were measured and interpreted as follows: penicillin (29 mm = sensitive in a higher dosage), erythromycin (32 mm = sensitive), rifampicin (50 mm = sensitive), meropenem (49 mm = sensitive), cotrimoxazole (30 mm = sensitive), ciprofloxacin (43 mm = sensitive in a higher dosage), linezolid (39 mm = sensitive) and tetracycline (46 mm = sensitive), respectively. In contrast to *C. ulcerans* and in line with the previously published data (9;14) the patient´s *C. silvaticum* strain was susceptible to clindamycin (26 mm).

**Fig. 3:**
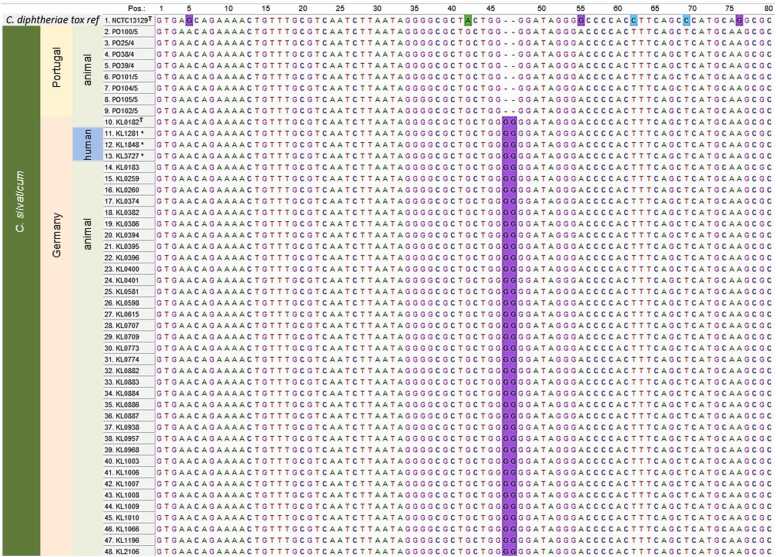
Excerpt of the first 80 nucleotides of the *tox* gene alignment of KL3727, the two other human *C. silvaticum* isolates and animal derived tox gene sequences from Clade 1 (Portugal) and Clade 2 (Germany), including *C. silvaticum* type strain KL0182^T^ and *C. diphtheriae* NCTC 13129 *tox* gene as reference. KL3727 and the two other human strains are marked with an asterisk.

NGS data analyses with cgMLST and *tox* gene alignment showed that KL3727 belongs to Clade 2. It showed the GG-insertion after position 46 in the *tox*-gene (Fig. 3), as all other so far sequenced Clade 2 *C. silvaticum* strains, including the two human-derived strains KL1281 and KL1848 [14]. This insertion of two nucleotides is leading to a frameshift and thereby to an inactive and immunologically undetectable DT protein.

**Fig. 4:**
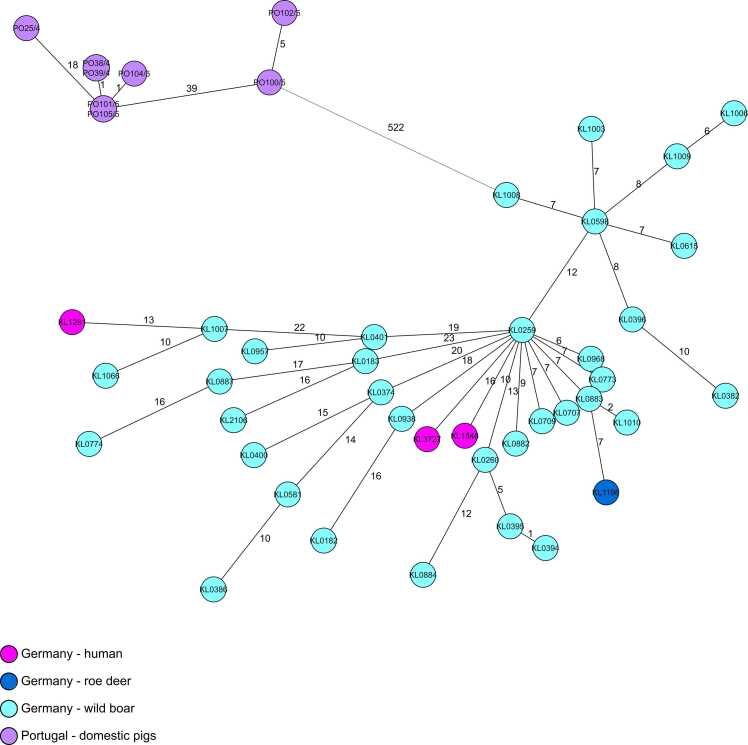
Minimum-spanning tree (MST) of an *C. silvaticum* core genome multilocus sequence typing scheme of 2012 target loci displaying KL3727, the two previously described human isolates KL1281 and KL1848, 35 animal-derived isolates from Germany as well as 8 isolates from clade 1 from Portugal. Allelic distances are indicated at connection lines.

cgMLST analysis also separates Clade 1, comprising the *tox*-positive Portuguese isolates and Clase 2, comprising KL3727 and all other German NTTB isolates, by more than 500 allelic differences (AD). There is no strict AD threshold that clearly delineates genetic relatedness in *C. silvaticum* and any thresholds always depend on the specific cgMLST scheme used. However, experiences with *C. ulcerans* show that epidemiologically confirmed direct transmission is usually characterized by no or very few genetic differences [1], [15], [16]. Our analysis therefore showed with minimum ADs of 13 that there is no close or direct genetic relationship between the three human isolates KL1281 (2018, Lower Saxony), KL1848 (2021, Northern Bavaria) and KL3727 (2024, Rhineland-Palatinate) or between human and animal isolates (Fig. 4).

After final identification as NTTB *C. silvaticum* antibiosis was switched to clindamycin 3 × 600 mg PO, the wound completely healed after the second surgical intervention, i.e. the exstirpation of the complete lymph node and abscess conglomerate, within a prolonged period of several weeks. Although his infection was caused by a NTTB strain the patient received a booster vaccination during reconvalescence by his private practitioner as recommended by the German Standing Committee on Vaccination (STIKO). His last anti-diphtheria booster immunization dated back more than the 10 years-period. No other *C. silvaticum* associated case was detected in his familiar or professional environment.

The patient is a passionate hunter who works in his own butcher's shop handling mainly wild boars, but also red deer, roe deer, fallow deer and mouflons after hunting events without wearing occupational safety gloves during slaughtering or animal processing. He reported to have experienced repeated microtraumas by gun-shot splintered animal bones especially on his hands and remembered to see regularly abscesses in the oral region of hunted animals. The patient was advised to use gloves for hunting and slaughtering procedures.

## Discussion

*C. silvaticum* [8] is a novel species originally isolated from forest-associated animals such as wild boars and roe deer, but recently also from a livestock pig [10], [11]. It has been delineated from the known zoonotic pathogen *C. ulcerans* for which animal-to-human transmission has been proven, e.g. from pet cats and dogs [1] and possibly also from livestock pigs [12]. Therefore, *C. silvaticum* has been suspected to be also a possible zoonotic member of the newly expanded *C. diphtheriae* complex. Very recently, two cases of human *C. silvaticum* infection linked to close animal contact (wild boar and dog) have been reported, both from rural areas in Germany (Northern Bavaria and Lower Saxony) [14]. While one of the cases, a butcher, remembered a concrete microtraumatizing event when field-dressing a hunted wild boar with suspicious mesenterial lymph nodes, the second infection affected a dog owner without close contact to wild forest-dwelling animals, making a transmission via the patient´s dog – most probably originating from a free living wood animal - the most plausible route of infection. The third human *C. silvaticum* case presented here also involved a person with both professional and recreational contact to animals remembering several occasions of microtraumas after processing slaughtered or hunted game animals, mostly wild boars, but also different deer species, some of them with obvious skin or mucosal ulcerations, with hands unprotected by gloves.

All 39 German isolates, including the three derived from human cases, were NTTB and belonged to Clade 2, in contrast to isolates from Portugal comprising another monophyletic clade of *tox*-positive *C. silvaticum* named Clade 1 and Austria where Clade 1 and Clade 2 occur [11], [14].

In terms of laboratory identification of *C. silvaticum*, its susceptibility to clindamycin gives a hint when differentiating *C. silvaticum* from clindamycin-resistant *C. ulcerans*, the most closely related species within the *C. diphtheriae* species complex. As in our case, antimicrobial susceptibility testing helped to guide antibiotic therapy when switching from perioperative empirical cefuroxime IV to oral clindamycin. Since commercial databases for MALDITOF-MS spectra analysis are often not yet able to differentiate between *C. ulcerans* and *C. silvaticum*, it might be useful to consider genetic methods for identifying *C. silvaticum*, esp.in the presence of clindamycin susceptibility and in isolates with a link to forest-dwelling animals such as wild boars and deer.

In conclusion, *C. silvaticum* should be considered as a potential zoonotic pathogen with possible animal-to-human transmission especially in hunters, butchers or rangers via direct contact to wild boars, deers, or infectious tissues from these animals. Suitable precaution measures to avoid the zoonotic risk of *C. silvaticum*, but also of *C. ulcerans* infection should be recommended when handling wild boars, pigs and deer.

## CRediT authorship contribution statement

**Robert B. Brauer:** Writing – original draft, Visualization, Methodology, Investigation, Conceptualization. **Anja Berger:** Writing – original draft, Supervision, Methodology, Investigation, Formal analysis, Conceptualization. **Andreas Sing:** Writing – original draft, Supervision, Funding acquisition, Conceptualization. **Katja Bengs:** Validation, Methodology, Investigation. **Hildegard Angermeier:** Validation, Methodology, Investigation. **Vyacheslav G. Melnikov:** Validation, Methodology, Investigation. **Alexandra Dangel:** Writing – original draft, Visualization, Methodology, Formal analysis.

## Informed consent

Written informed consent was obtained from the patient for publication of this case report and any accompanying images or clinical details. A copy of the written consent is available for review by request.

## Ethical approval

Written consent was obtained from the patient to publish this case report and all accompanying clinical data and images.

## Declaration of Generative AI and AI-assisted technologies in the writing process

There was no use of a chatbot or artificial intelligence tool for any portion of your work.

## Role of the funding source

The study was partly supported by the Bavarian State Ministry of Health, Care and Prevention as well as by the German Federal Ministry of Health via the Robert Koch-Institute (09–47, FKZ 1369-359).

## Declaration of Competing Interest

The authors declare that there are no financial interests/personal relationships which may be considered as potential competing interests.

## Data Availability

All relevant data from the clinical case are included in this article. NGS raw data of KL3727 is available at National Center for Biotechnology Information Short Read Archive (http://www.ncbi.nlm.nih.gov/sra) at Bioproject PRJNA1205636 with BioSample accession SAMN51352217.
